# LINC00160 mediates sunitinib resistance in renal cell carcinoma via SAA1 that is implicated in STAT3 activation and compound transportation

**DOI:** 10.18632/aging.103755

**Published:** 2020-09-13

**Authors:** Gong Cheng, Yuenan Liu, Lilong Liu, Hailong Ruan, Qi Cao, Zhengshuai Song, Lin Bao, Tianbo Xu, Zhiyong Xiong, Jingchong Liu, Di Liu, Huageng Liang, Guosong Jiang, Xiong Yang, Hongmei Yang, Ke Chen, Xiaoping Zhang

**Affiliations:** 1 Department of Urology, Union Hospital, Tongji Medical College, Huazhong University of Science and Technology, Wuhan 430022, China; 2 Institute of Urology, Union Hospital, Tongji Medical College, Huazhong University of Science and Technology, Wuhan 430022, China; 3 Department of Pathogenic Biology, School of Basic Medicine, Tongji Medical College, Huazhong University of Science and Technology, Wuhan 430022, China

**Keywords:** LINC00160, SAA1, renal cell carcinoma, sunitinib, drug resistance

## Abstract

Patients with advanced renal cell carcinoma who are resistant to sunitinib currently have limited clinical options for treatment. Therefore, it is necessary to explore the biological basis of sunitinib resistance and to uncover new targets for the intervention of sunitinib resistance. In this study, we identified that LINC00160 was associated with sunitinib resistance in renal cell carcinoma. Resistant tumor cells highly expressed LINC00160 to recruit transcriptional factor TFAP2A, which bound to SAA1 promoter regions and activated its expression. On one hand, SAA1 linked to ABCB1 protein, which facilitated sunitinib cellular efflux and diminished drug accumulation. On the other hand, SAA1 stimulated JAK-STAT signaling pathways, which countered cellular survival inhibition from drug. All these regulatory networks were well organized and collaborated, thus promoting sunitinib resistance in renal cell carcinoma. LINC00160 mediates sunitinib resistance in renal cell carcinoma via SAA1 that is implicated in STAT3 activation and compound transportation, which offers an opportunity for targeted intervention and molecular therapies in the future.

## INTRODUCTION

Renal cell carcinoma (RCC) is one of the most common malignancies worldwide, accounting for about 3-5% of the estimated male mortality [1]. Clear cell renal cell carcinoma (ccRCC) is the most common type of RCC, which is characterized by inactivating mutations in the VHL gene [2] and is associated with a high risk of metastasis and poor response to chemotherapy [3]. Although surgeries are preferred for localized renal cell carcinoma, comprehensive treatments are more important in metastatic renal cell carcinoma (mRCC) [4, 5]. Recent advances in understanding of RCC pathogenesis has led to the development of targeted agents, such as sunitinib [6]. However, part of RCC patients have no response to sunitinib therapy, and ultimately end up with drug resistance and disease progression [7].

Non-coding RNAs (ncRNAs) are transcripts without protein coding potential, playing a crucial role in various cellular and physiologic processes [8–10]. Long ncRNAs (lncRNAs), which are >200 nucleotides long, have recently been shown to exhibit various modulatory roles during tumorigenesis, which includes recruitment of transcriptional factors by acting as modular scaffolds and interactions with miRNAs, mRNAs or proteins at post-transcriptional regulatory level [11–14]. Dysregulated expression patterns of lncRNAs in cancer development indicate that these lncRNAs may act as potential molecular biomarkers [15]. In addition, recent studies have discovered connections between drug resistance and non-mutational modulation of gene expression, during which lncRNAs could function as major modulators and affect drug sensitivity to cancer cells [16, 17]. Up till now, few evidences have indicated that lncRNAs participate in sunitinib resistance of RCC [6, 18]. Considering the highly specific phenotype of cancer types, it is necessary to investigate intrinsic mechanisms and find key genes regulating signaling pathways in sunitinib resistance, which provides opportunities for early intervention and therapeutic targets for cancer treatment.

Serum amyloid A1 (SAA1) is an acute-phase protein that is highly expressed in response to inflammation and tissue damage [19]. Significant overexpression of SAA1 has been observed in various types of cancer and directly correlates with poor prognosis and tumor progression [20, 21]. A proteomic analysis of serum and tissue samples from RCC patients revealed that SAA1 levels were associated with tumor stage and metastasis, which indicates that SAA1 acts as a potential specific biomarker for aggressive clear cell RCC [22–25]. Twenty six serum samples from mRCC patients prior and after sunitinib therapy were analyzed by mass spectrometry [26]. High SAA1 expression levels are representative for patients with poor response to sunitinib, which indicates that SAA1 could exhibit protein signatures for tyrosine kinase inhibitor therapy response prediction and might participate in sunitinib resistance of RCC patients.

In the previous study, we found that lncRNA-LINC00160 acted as an oncogenic gene and exhibited diagnostic and prognostic values in renal cell carcinoma after screening from cancer progression-related gene sets and sunitinib resistance-related gene sets. In this study, we further explored that LINC00160 was overexpressed in sunitinib-resistant RCC cells. Moreover, experiments conducted both *in vitro* and *in vivo* models have revealed that LINC00160 recruited transcriptional factor AP-2 alpha (TFAP2A), which bound to serum amyloid A1 (SAA1) promoter regions and activated its expression. On one hand, SAA1 anchored to ATP binding cassette subfamily B member 1 (ABCB1) protein, which facilitated sunitinib cellular efflux and diminished drug accumulation. On the other hand, SAA1 stimulated JAK-STAT signaling pathways, which countered cellular survival inhibition from drug. These findings provide a new understanding of sunitinib resistance in renal cell carcinoma, which could pave the way for targeted intervention and molecular therapies in the future.

## RESULTS

### Establishment of RCC sunitinib resistant cell lines

**Figure 1:**
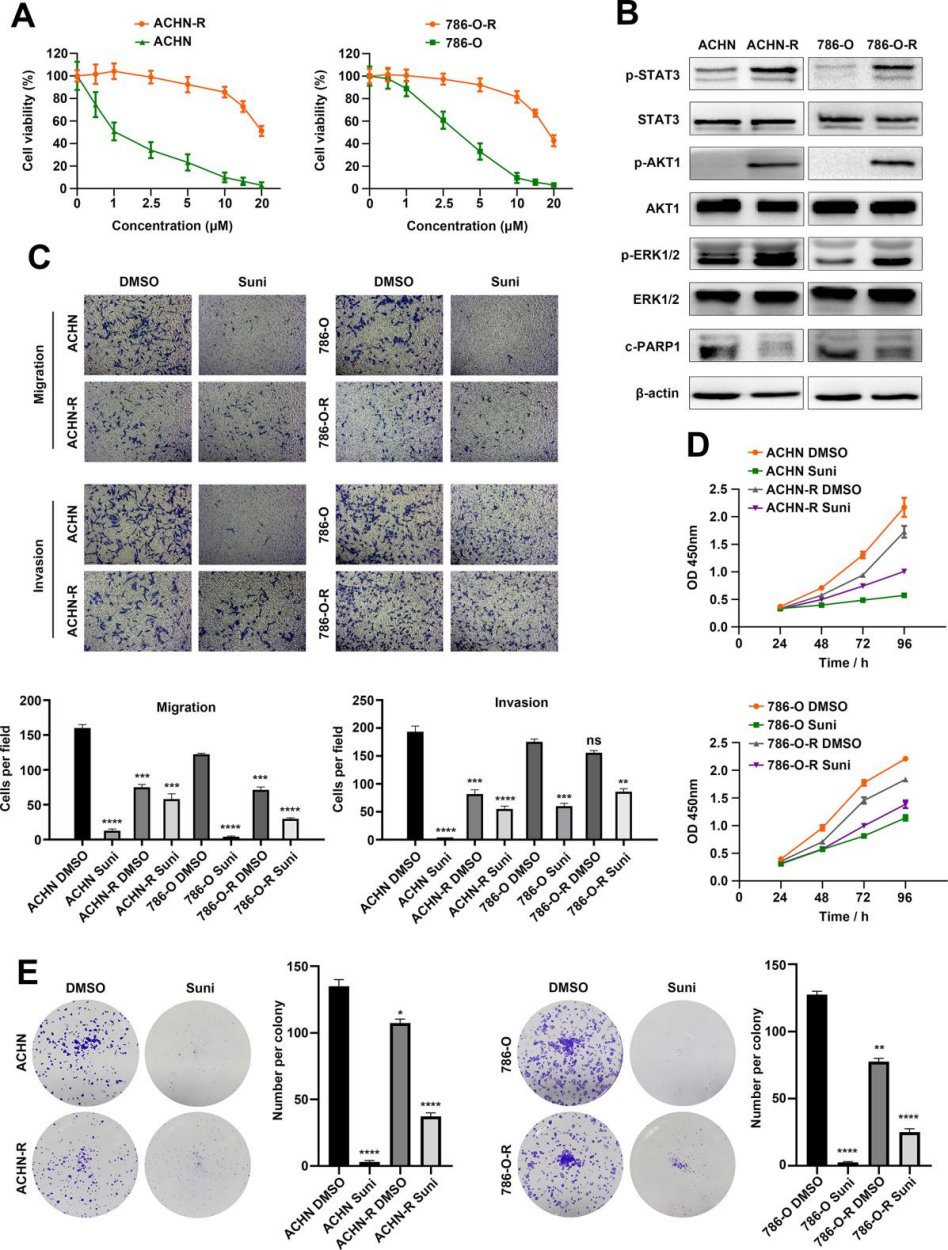
**Establishment of RCC sunitinib resistant cell lines.** (**A**) Cell viability of resistant cells ACHN-R, 786-O-R and parental cells ACHN, 786-O in sunitinib concentration gradients. (**B**) Western blotting analysis of c-PARP1and phosphorylated and total STAT3, AKT1 and ERK1/2 after sunitinib treatment in resistant and parental cell lines. β-actin served as the loading control. (**C**) Transwell assays of resistant and parental cells with/without sunitinib treatment. (**D**) CCK8 assays of resistant and parental cells with/without sunitinib treatment. (**E**) Colony formation of resistant and parental cells with/without sunitinib treatment. Each experiment was performed at least three times and data was represented as mean ± SEM. *P<0.05, **P<0.01, ***P<0.001 and ****P<0.0001. RCC, renal cell carcinoma; c-PARP1, cleaved poly(ADP-ribose) polymerase 1; STAT3, signal transducer and activator of transcription 3; AKT1, AKT serine/threonine kinase 1; ERK1/2, mitogen-activated protein kinase 3/1; CCK8, cell counting kit-8.

Sunitinib resistant cell lines ACHN-R and 786-O-R were established by continuously exposing ACHN and 786-O cells to sunitinib environment [27]. Examination of cell morphology revealed that sunitinib resistant cells were more flattened and stretched as compared with parental cells (Supplementary Figure 1A). We then exposed resistant and parental cells to sunitinib environment with concentration gradients and cell viability assays revealed that resistant cells exhibited much higher tolerance to sunitinib therapy (Figure 1A). Growing evidences have illustrated that activation of alternative survival signaling pathways might contribute to therapeutic resistance [28] and we tested three classic survival signaling pathways via Western blotting. Proliferation-associated proteins p-STAT3, p-AKT1 and p-ERK1/2 were inhibited after sunitinib treatments, while apoptosis-related protein c-PARP1 was at higher expression levels in ACHN and 786-O cells (Figure 1B). Transwell assays were performed to assess cell migration and invasion abilities with/without sunitinib treatment. As shown in Figure 1C, parental cells presented obvious potential than resistant cells without drug therapy. However, this phenomenon was reversed after cells exposed to sunitinib environment. Results indicated that resistant cells were less sensitive to sunitinib and restriction was weakened to motility of cells. Similar phenomenon was observed in wound healing assays (Supplementary Figure 1B). Parental cells migrated at a higher speed than resistant cells with no drug exposure, but slowed down after treatment with sunitinib. We further compare proliferation rate between parental and resistant cells. As shown in Figure 1D, parental cells grown faster than resistant cells without drug exposure, but were inhibited after sunitinib treatment. Resistant cells exhibited less sensitivity to sunitinib compared with parental cells and therefore continued to grow in drug environment. This result was also verified with colony assays (Figure 1E). Based on these findings, we believed that sunitinib resistant RCC cells were established which met the demand of further researches.

### LINC00160 participates in sunitinib resistance of RCC

**Figure 2:**
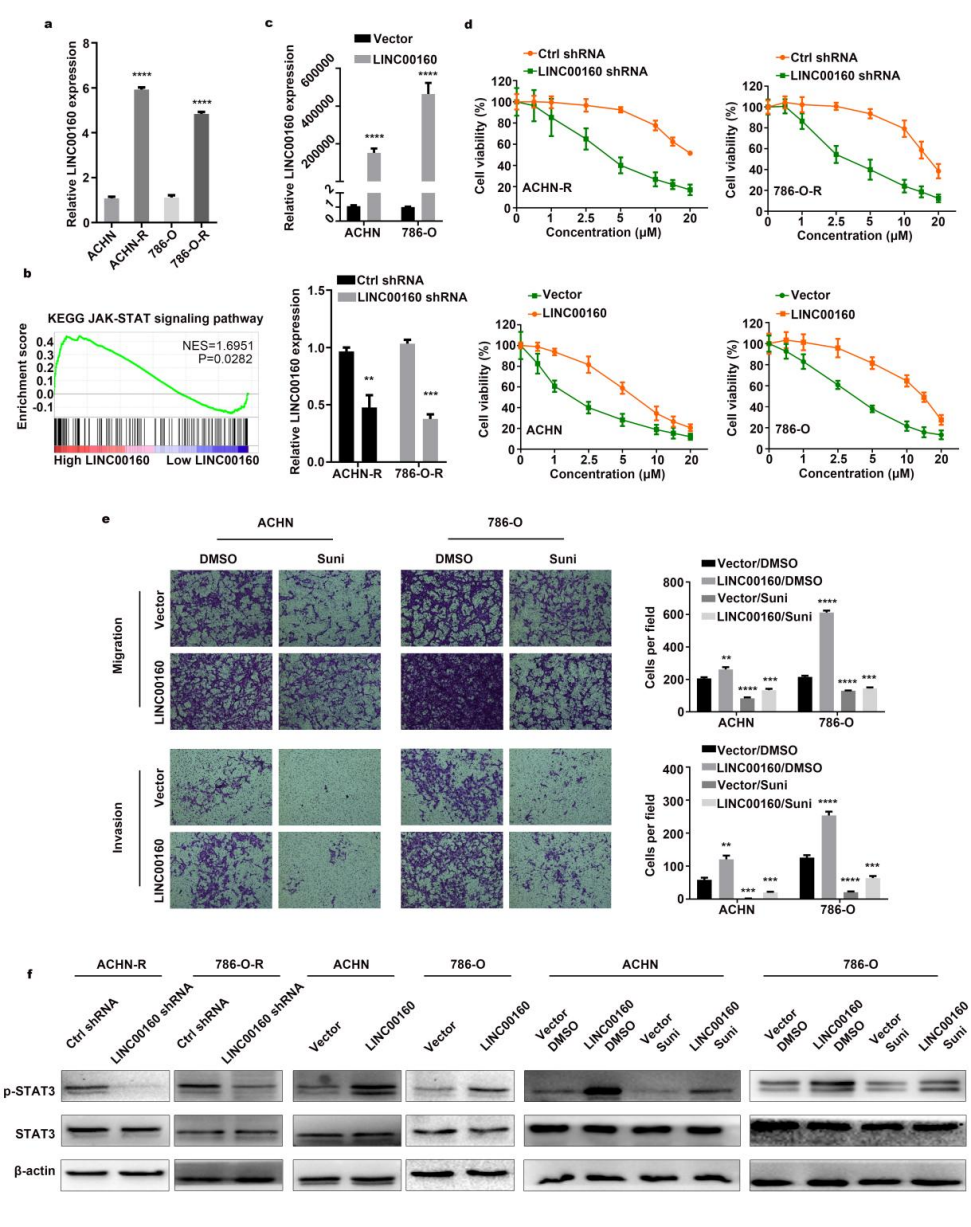
**LINC00160 participates in sunitinib resistance of RCC.** (**A**) LINC00160 expression was compared between resistant and parental cells by RT-qPCR. (**B**) GSEA analysis revealed that high LINC00160 expression was involved in JAK-STAT signaling pathway. (**C**) LINC00160 expression was detected by RT–qPCR after knockdown and overexpression. (**D**) Cell viability assays in sunitinib concentration gradients were conducted after LINC00160 knockdown and overexpression. (**E**) Transwell assays were conducted after sunitinib treatment and LINC00160 overexpression. (**F**) Western blotting analysis of p-STAT3 and STAT3 after silencing LINC00160 in resistant cells, upregulating LINC00160 in parental cells and overexpressing LINC00160 combined with sunitinib treatment in parental cells. β-actin served as the loading control. Each experiment was performed at least three times and data was represented as mean ± SEM. *P<0.05, **P<0.01, ***P<0.001 and ****P<0.0001. LINC00160, long non-coding RNA 160; RCC, renal cell carcinoma; RT-qPCR, quantitative real time polymerase chain reaction; GSEA, gene set enrichment analysis; JAK, Janus kinase; p-STAT3, phosphorylated signal transducer and activator of transcription 3; STAT3, signal transducer and activator of transcription 3.

As LINC00160 was selected from sunitinib resistance-related gene sets, we wanted to explore whether LINC00160 was truly participate in the resistance process. LINC00160 expression was upregulated in resistant cells 5-times higher than parental cells (Figure 2A). Gene set enrichment analysis (GSEA) was also conducted, which indicated that higher LINC00160 expression was enriched in JAK-STAT signaling pathway (Figure 2B). After knocking down LINC00160 and overexpressing LINC00160 expression (Figure 2C), we evaluated cell viability in sunitinib concentration gradients. Compared with control group, resistant cells were sensitized to sunitinib after downregulating LINC00160 expression (Figure 2D). Tolerance traits were slightly elevated with higher LINC00160 expression level in RCC cells. To further verify conferred tolerance to sunitinib after upregulating LINC00160, we designed transwell rescuing assays with sunitinib treatment. As shown in Figure 2E, parental cells with high LINC00160 expression level and no drug treatment migrated and invaded the most, followed by the control group cells with no external intervention. Cells exposed to sunitinib environment exhibited the worst motility while overexpressing LINC00160 could enhance cells motility after drug treatment. To verify the prediction of GSEA results, cells with intervention were lysed for protein extraction. Decreased level of p-STAT3 were observed in downregulated LINC00160 groups as compared to control groups in resistant cells (Figure 2F). Increased level of p-STAT3 were found in upregulated LINC00160 groups when compared to control groups in parental cells. The highest level of p-STAT3 was observed in LINC00160 overexpression groups with no drug therapy, while the lowest level of p-STAT3 was found in sunitinib treatment groups. However, upregulation of LINC00160 could elevated the expression level of p-STAT3 and partially reversed inhibitory effects of drug. Therefore, we believed that RCC cells exhibited tolerance to drug via upregulating LINC00160 expression and thus activating JAK-STAT signaling pathway.

### SAA1 is a candidate downstream molecule of LINC00160 in RCC cells

**Figure 3:**
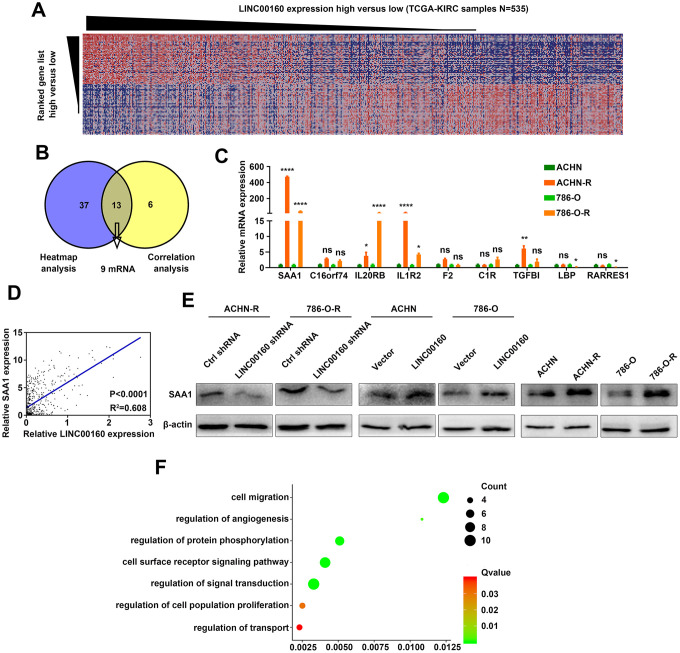
**SAA1 is a candidate downstream molecule of LINC00160 in RCC cells.** (**A**) Heatmap analysis of TCGA-KIRC samples (N=535) ranking LINC00160 expression from high to low. (**B**) Candidate mRNAs selected from GSEA analysis and correlation analysis. (**C**) Candidate genes expressions were verified in resistant and parental cells by RT-qPCR. (**D**) Correlation between SAA1 and LINC00160 expression. (**E**) Western blotting analysis of SAA1 after silencing LINC00160 in resistant cells, upregulating LINC00160 in parental cells and comparison between parental and resistant cells. β-actin served as the loading control. (**F**) GO analysis of SAA1 in enrichment pathways. Enrichment pathways of SAA1 after GO analysis. Each experiment was performed at least three times and data was represented as mean ± SEM. *P<0.05, **P<0.01, ***P<0.001, ****P<0.0001 and P>0.05 is denoted by ns. SAA1, serum amyloid A1; LINC00160, long non-coding RNA 160; RCC, renal cell carcinoma; TCGA-KIRC, the cancer genome atlas program- kidney renal clear cell carcinoma; GSEA, gene set enrichment analysis; RT-qPCR, quantitative real time polymerase chain reaction; GO, gene ontology.

To figure out which proteins were directly regulated by LINC00160, heatmap analysis was conducted based on GSEA results (Figure 3A). Positively correlated genes were selected from the TCGA-KIRC database and 9 protein coding genes were found from heatmap analysis and correlation analysis (Figure 3B). We compared these 9 mRNA expression levels between resistant and parental cells and found that SAA1 exhibited the highest expression level in sunitinib resistant cells (Figure 3C and Supplementary Figure 2). Correlation between LINC00160 and SAA1 in TCGA-KIRC samples (n=535) was strong (R^2^=0.608 with statistically significant difference) (Figure 3D). We further conducted experiments in protein levels and SAA1 was downregulated after LINC00160 knockdown in resistant cells and upregulated after LINC00160 overexpression in parental cells (Figure 3E). SAA1 expression was elevated in sunitinib resistant cells compared to parental cells. Gene ontology (GO) analysis was made and higher SAA1 expression was enriched in signaling pathways, such as cell migration, regulation of angiogenesis, regulation protein phosphorylation, cell surface receptor signaling pathway, regulation of cell population proliferation and regulation of transport (Figure 3F). Based on these results, we presumed that SAA1 was a candidate downstream molecule of LINC00160 in RCC cells.

### SAA1 mediates sunitinib resistance of RCC

**Figure 4:**
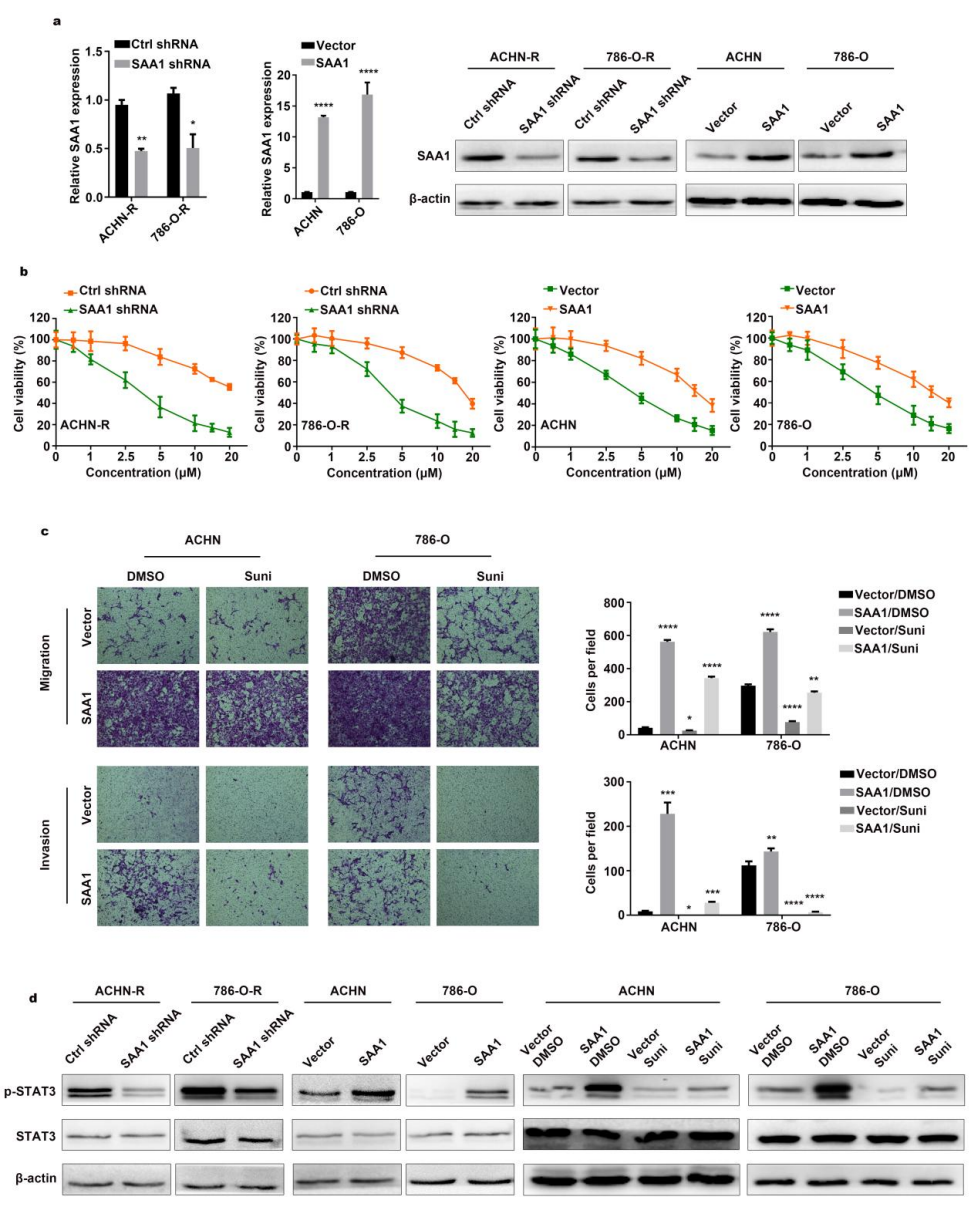
**SAA1 mediates sunitinib resistance of RCC.** (**A**) SAA1 expression was detected by RT–qPCR and western blotting after knockdown and overexpression. (**B**) Cell viability assays in sunitinib concentration gradients were conducted after SAA1 knockdown and overexpression. (**C**) Transwell assays were conducted after sunitinib treatment and SAA1 overexpression. (**D**) Western blotting analysis of p-STAT3 and STAT3 after silencing SAA1 in resistant cells, upregulating SAA1 in parental cells and overexpressing SAA1 combined with sunitinib treatment in parental cells. β-actin served as the loading control. Each experiment was performed at least three times and data was represented as mean ± SEM. *P<0.05, **P<0.01, ***P<0.001 and ****P<0.0001. SAA1, serum amyloid A1; RCC, renal cell carcinoma; RT-qPCR, quantitative real time polymerase chain reaction; p-STAT3, phosphorylated signal transducer and activator of transcription 3; STAT3, signal transducer and activator of transcription 3.

After knocking down SAA1 and overexpressing SAA1 expression (Figure 4A), we evaluated cell viability in sunitinib concentration gradients. Compared with control groups, resistant cells were sensitized to sunitinib after downregulating SAA1 expression (Figure 4B). Tolerance traits were slightly elevated with higher SAA1 expression level in RCC cells. To further verify conferred tolerance to sunitinib after upregulating SAA1, we designed transwell rescuing assays with sunitinib treatment. As shown in Figure 4C, parental cells with high SAA1 expression level and no drug treatment migrated and invaded the most, followed by the control group cells with no external intervention. Cells exposed to sunitinib environment exhibited the worst motility, while overexpressing SAA1could enhance cells motility after drug treatment. To verify whether SAA1 mediated JAK-STAT signaling pathway, cells with intervention were lysed for protein extraction. Decreased level of p-STAT3 were observed in downregulated SAA1 groups as compared to control groups in resistant cells (Figure 4D and Supplementary Figure 3). Increased level of p-STAT3 were found in upregulated SAA1 groups when compared to control groups in parental cells. The highest level of p-STAT3 was observed in SAA1 overexpression groups with no drug therapy, while the lowest level of p-STAT3 was found in sunitinib treatment groups. Besides, upregulation of SAA1 could elevated the expression level of p-STAT3 and partially reversed inhibitory effects of drug. Therefore, we believed that RCC cells exhibited tolerance to drug via upregulating SAA1 expression and thus activating JAK-STAT signaling pathway.

### Regulatory networks between LINC00160 and SAA1 in RCC cells

**Figure 5:**
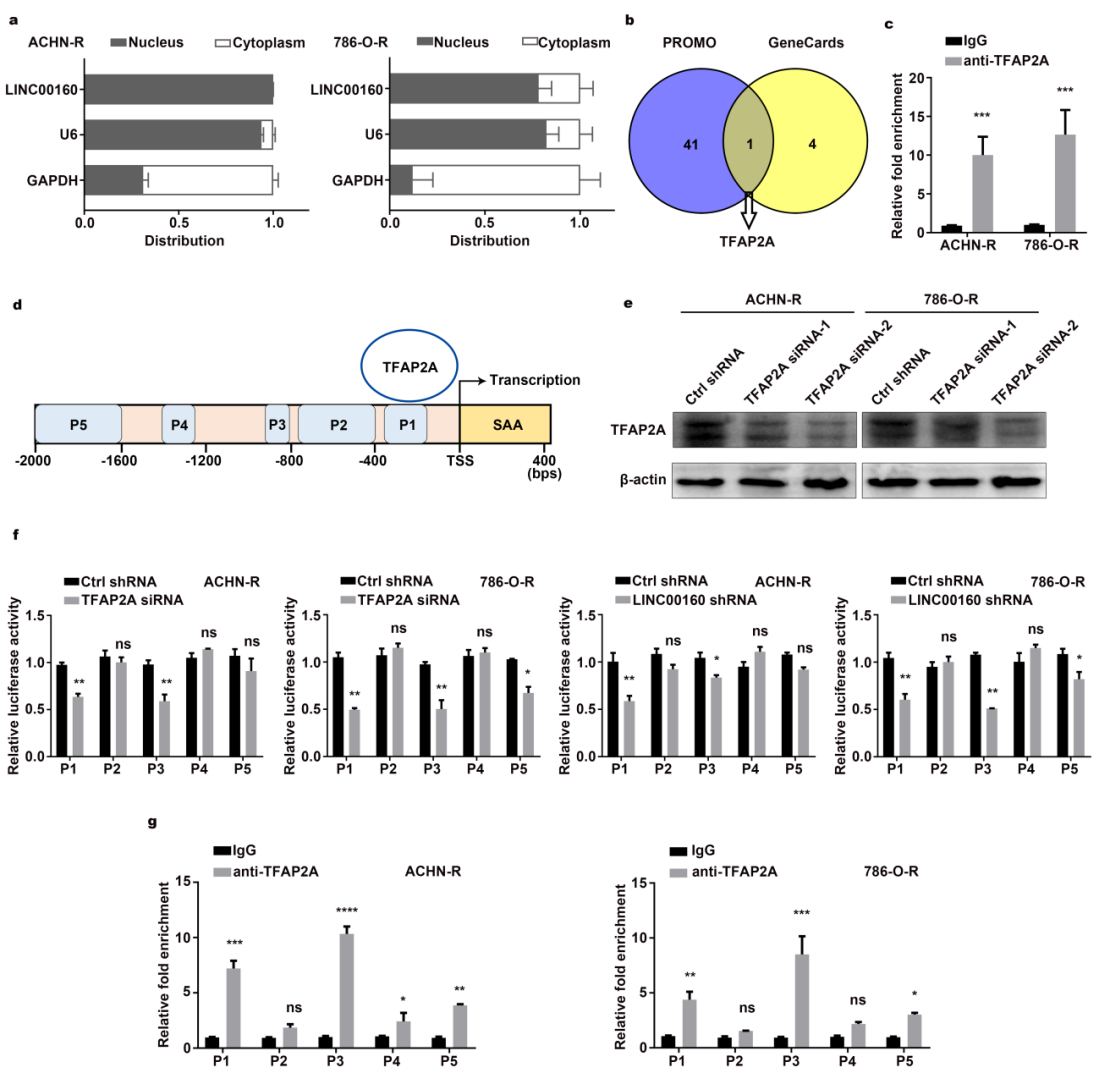
**Regulatory networks between LINC00160 and SAA1 in RCC cells.** (**A**) RT-qPCR analysis of LINC00160 in the subcellular fractions of resistant cells. U6 and GAPDH acted as nuclear and cytoplasmic markers respectively. (**B**) Transcriptional factors were selected from PROMO and GeneCards databases. (**C**) RNA immunoprecipitation (RIP) experiments were performed using anti-TFAP2A antibody in resistant cells. (**D**) 5 predicted bound sites of SAA1 promoter region. (**E**) Western blotting analysis of TFAP2A after knockdown. β-actin served as the loading control. (**F**) Luciferase activity assays of SAA1 promoter regions in resistant cells after LINC00160 and TFAP2A knockdown respectively. (**G**) Chromatin immunoprecipitation (CHIP) assay of TFAP2A enrichment at SAA1 promoter region relative to control IgG in resistant cells. Each experiment was performed at least three times and data was represented as mean ± SEM. *P<0.05, **P<0.01, ***P<0.001 and ****P<0.0001. P>0.05 is denoted by ns. LINC00160, long non-coding RNA 160; SAA1, serum amyloid A1; RCC, renal cell carcinoma; RT-qPCR, quantitative real time polymerase chain reaction; U6, RNA U6 Small Nuclear 1; GAPDH, glyceraldehyde-3-phosphate dehydrogenase; RIP, RNA immunoprecipitation; TFAP2A, transcription factor AP-2 alpha; CHIP, chromatin immunoprecipitation.

LncRNAs exhibit various modulatory roles through different mechanisms, largely depending on subcellular localization [29, 30]. Separation of nuclear and cytoplasmic RNA assays revealed that LINC00160 was predominantly localized in the nucleus rather than in the cytoplasm of resistant cells (Figure 5A). Previous studies have reported that lncRNAs may act as modular scaffolds to recruit transcriptional factors and regulate gene expression [14]. To explore the regulatory network between LINC00160 and SAA1 in sunitinib resistant cells, we sought to identify LINC00160 binding factors. PROMO and GeneCards websites were screened and transcriptional factor for SAA1 was selected (Figure 5B). RNA Immunoprecipitation (RIP) assays were conducted and anti-TFAP2A antibody was used to precipitate endogenous TFAP2A from nuclear extracts (Figure 5C). RNA bound to proteins was extracted and LINC00160 was approximately 10-fold enrichment in the anti-TFAP2A immunoprecipitation, compared with the IgG control. To identify whether TFAP2A exerted effects on SAA1 gene expression, we designed 5 fragments in SAA1 promoter regions (namely P1, P2, P3, P4, P5) (Figure 5D). After knocking down TFAP2A (Figure 5E), we constructed SAA1 dual-luciferase reporters. Luciferase activities of P1 and P3 sites were weakened with lower TFAP2A expression (Figure 5F and Supplementary Figure 4). Similar results were found after silencing LINC00160 in resistant cells, which indicated that LINC00160 might recruit TFAP2A to bind P1 and P3 sites of SAA1 promoter regions. Chromatin immunoprecipitation assay and promoter analysis (CHIP) assays were also conducted to validate our assumption. As shown in Figure 5G, TFAP2A bound to P1, P3 and P5 sites of SAA1 promoter regions with 5, 10 and 2-fold enrichment respectively as compared with the IgG control. All these have indicated that LIC00160 acted as scaffolds to recruit TFAP2A, which mainly bound to P1 and P3 sites of SAA1 promoter regions and activating SAA1 gene expression at transcriptional level.

### ABCB1 facilitates drug efflux of RCC cells

**Figure 6:**
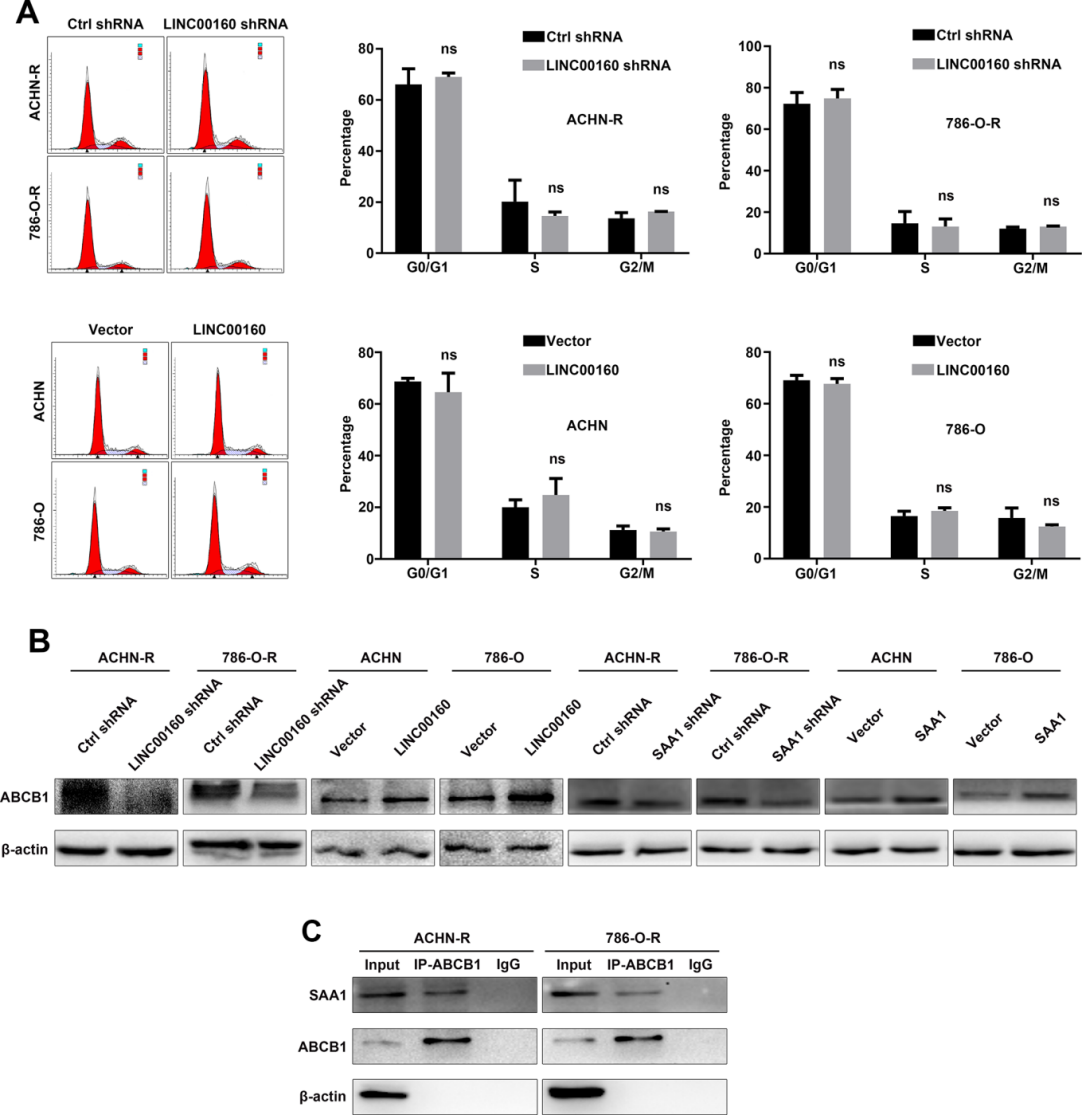
**ABCB1 facilitates drug efflux of RCC cells.** (**A**) Cell cycles were detected in resistant cells after LINC00160 knockdown and overexpression. (**B**) Western blotting analysis of ABCB1 after silencing LINC00106 and SAA1 in resistant cells respectively, upregulating LINC00160 and SAA1 in parental cells respectively. β-actin served as the loading control. (**C**) Resistant cell lysate was subjected to immunoprecipitation with anti-ABCB1 antibody and analyzed by western blotting. Each experiment was performed at least three times and data was represented as mean ± SEM. P>0.05 is denoted by ns. ABCB1, ATP binding cassette subfamily B member 1; RCC, renal cell carcinoma; LINC00160, long non-coding RNA 160; SAA1, serum amyloid A1.

New researches have revealed that neoplastic cells transition into a quiescent state with expansion of G0 gap when exposed to stressors, and keep tolerance to drug therapy but do not proliferate [31]. This finding raised our interests and we conducted flow cytometry analysis to investigate whether LINC00160 participated in cell cycle process to mediate drug resistance. As shown in Figure 6A, downregulation of LINC00160 did not arrest cells in G0/G1 or G2/M gap, while overexpressing LINC00160 had no effects on transiting resistant cells from G0/G1 gap into S phase. These results indicated that LINC00160 exerted no effects on regulation of cell cycles in resistant cells.

It has been reported that ABCB1 participates in the accumulation of sunitinib in autolysosomes and favors its cellular efflux, thus leading to drug resistance of RCC cells [32]. Based on these findings, we wanted to explore whether LINC00160/SAA1 axis could regulate ABCB1 protein to facilitate drug efflux. ABCB1 expression was decreased after knocking down LINC00160 or SAA1 expression in resistant cells (Figure 6B). ABCB1 expression was elevated with higher LINC00160 or SAA1 expression in parental cells. Since SAA1 was predicted to regulate transport base on GO analysis, we wanted to uncover the interaction between these two proteins. Immunoprecipitation assays were applied and anti-ABCB1 antibody was used to precipitate ABCB1 protein. SAA1 protein was also pulled down from cell lysate as compared with the IgG control (Figure 6C). Therefore, we believed that SAA1 bound to ABCB1 protein, which facilitated sunitinib efflux and diminished drug accumulation in RCC cells.

### Combined targeted therapy simulated *in vivo*

**Figure 7:**
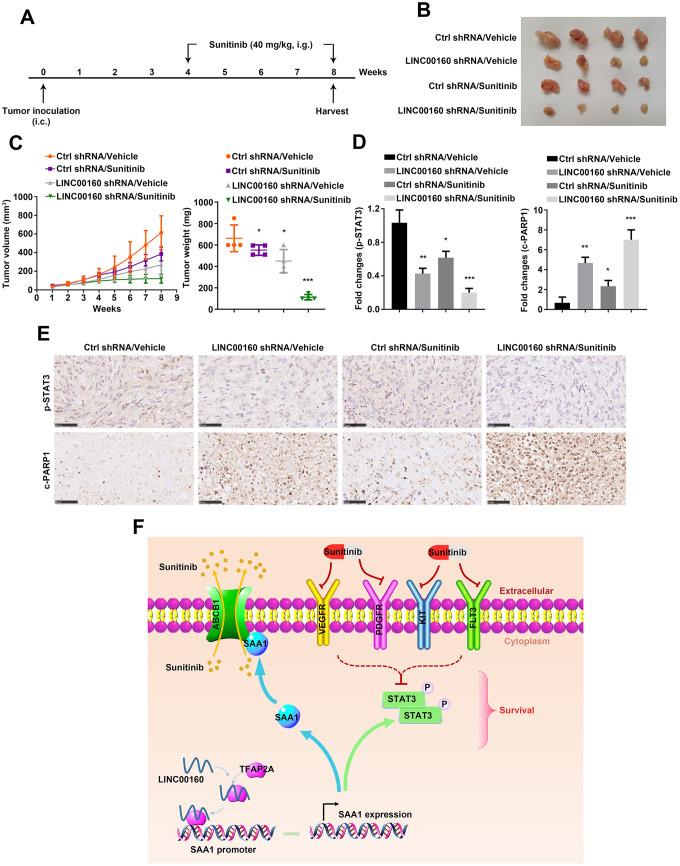
**Combined targeted therapy simulated *in vivo*.** (**A**) A schematic diagram of subcutaneous xenograft schedule. (**B**) LINC00160 knockdown 786-O-R cells were subcutaneously injected into the flanks of nude mice. Xenografts were harvested after 8 weeks. (**C**) Tumor weight was measured after resection. Tumor volume was calculated every week. (**D**, **E**) Immunohistochemistry staining for p-STAT3 and c-PARP1 were examined. Black scale bar represents 50 μm (**E**). Fold changes were presented (**D**). (**F**) A schematic diagram indicated LINC00160 regulatory network during resistant process. Data was represented as mean ± SEM. *P<0.05, **P<0.01 and ***P<0.001. LINC00160, long non-coding RNA 160; p-STAT3, phosphorylated signal transducer and activator of transcription 3; c-PARP1, cleaved poly (ADP-ribose) polymerase 1.

It has been shown that alteration of LINC00160 expression level influenced cell motility in *vitro*, we wanted to further validate its effects on tumor growth *in vivo*. As shown in Figure 7A, 786-O-R cells were implanted subcutaneously into nude mice flanks. After 8 weeks, knocking down LINC00160 remarkably suppressed tumor growth and this effect was reinforced by combination with sunitinib therapy (Figure 7B, 7C). Tumor samples collected from xenografts were undertaken immunohistochemistry (IHC). It has shown that p-STAT3 protein was highest expressed with no external interference (Figure 7D, 7E). Silencing LINC00160 would suppress JAK-STAT signaling pathways and this inhibitory effect was strengthened combined with LINC00160 knockdown and sunitinib treatment. However, c-PARP1 expression levels in four groups were conversed to p-STAT3 expression. The highest level of c-PARP1 was observed in LINC00160 silencing plus drug therapy group, while the lowest level of c-PARP1 was found in the normal control group.

Conclusively, we summarized our research with a schematic diagram (Figure 7F). During sunitinib resistance processes, RCC cells highly expressed LINC00160 to recruit transcriptional factor-TFAP2A. TFAP2A bound to SAA1 promoter regions and activated its expression. On one hand, SAA1 anchored to ABCB1 protein, which facilitated sunitinib cellular efflux and diminished drug accumulation. On the other hand, SAA1 stimulated JAK-STAT signaling pathways, which countered cellular survival inhibition from drug. All these regulatory networks were well organized and collaborated, thus promoting sunitinib resistance in renal cell carcinoma.

## DISCUSSION

Advanced RCC patients currently have limited clinical options for treatment when resistant to targeted therapy. Therefore, it is necessary to explore the inner mechanism of sunitinib resistance and to uncover new targets for intervention. LINC00160 has been reported to be transcriptional targets of ERα and exhibit prognostic significance in breast cancer survival [33, 34]. Additionally, downregulation of LINC00160 resulted in suppression of cancer cells proliferation, which indicated that LINC00160 could act as a biomarker for endocrine response in breast cancer therapy. In this study, we identified LINC00160 to be highly expressed in sunitinib resistant cells. Bioinformatic analysis revealed that LINC00160 involved in JAK-STAT signaling pathway. JAK-STAT signaling pathway was closely associated with cell survival and could partially reflect cell viability in certain circumstances. After upregulating LINC00160 expression, JAK-STAT signaling pathways was stimulated and drug tolerance traits were enhanced in RCC cells. Downregulation of LINC00160 attenuated p-STAT3 expression and restored drug sensitivity in sunitinib resistant cells. AKT/ERK were also classical survival signaling pathways apart from JAK-STAT. In our studies, upregulation of LINC00160 slightly stimulated p-AKT1 and p-ERK1/2 expression, but the results exhibited no statistical difference. Therefore, we have focused our attention to JAK-STAT signaling pathway and explored regulatory mechanism after drug treatment. ABCB1 involves in multidrug resistance and acts as an ATP-dependent drug efflux pump for xenobiotic compounds with broad substrate specificity [35]. It is responsible for decreased drug accumulation in multidrug-resistant cells and often mediates the development of resistance to anticancer drugs. It has been revealed that when sunitinib was trapped in lysosomes and localized to the cytoplasm, sunitinib was transported out of the cell by the ABCB1 proteins, which further leaded to drug tolerance [32]. However, regulation of ABCB1 in sunitinib resistant cells was unclear and we found that LINC00160 could stimulate ABCB1 expression to attenuate cytotoxicity of the drug.

SAA1 has been reported to function as a biomarker for advanced renal cell carcinoma [25]. Patients with poor response after tyrosine kinase inhibitors (TKIs) treatment exhibited significant SAA1 expression level, which indicated SAA1 might participate in sunitinib resistance [26]. Functional analysis has revealed that SAA1 mediated RCC cells poor response to sunitinib. Since SAA1 was predicted to involve regulation of transport, we conducted immunoprecipitation assays and found that SAA1 could anchored to ABCB1 to facilitate drug efflux. As for detailed regulatory networks between LINC00160 and SAA1, transcriptional factor-TFAP2A was introduced and mainly bound to -360/-164 base pairs (bp) sites and -1442/-1309 bp sites of SAA1 promoter region, thus regulating SAA1 gene expression at transcriptional level.

Targeted therapy is an important method for advanced renal cell carcinoma, however acquired or adaptive resistance is inevitable during the treatment processes. Different pathogenesis occurred when tumors exhibited primary refractoriness to first-line TKI treatment. Established hypothesis included revascularization consequent to upregulation of alternative pro-angiogenic signals; protection of the tumor vasculature either by recruiting pro-angiogenic inflammatory cells or by increasing protective pericyte coverage; accentuated invasiveness of tumor cells into local tissue to co-opt normal vasculature; and increased metastatic seeding and tumor cell growth in lymph nodes and distant organs [36]. Up till now, few researches have been focused on lncRNAs roles in sunitinib resistance of renal cell carcinoma. LncRNA-SARCC has been reported to be associated with better prognosis in RCC. Increased expression of LncRNA-SARCC followed by sunitinib treatment would attenuate RCC cells resistance to sunitinib [18]. LncRNA-lncARSR, which correlated with poor sunitinib response, mediated sunitinib resistance via sponging miR-34/miR-449 to facilitate AXL and c-MET expression in RCC cells. Besides, lncARSR could be incorporated into exosomes and transferred to sunitinib sensitive cells, thus disseminating drug resistance [6]. Some researchers have pointed that cancer cells often reprogram their metabolic pathways to adapt to environmental challenges and facilitate survival, proliferation, and metastasis. It seems possible that the progression of renal cancer is closely related to glycolysis and metabolic processes [15]. Growth inhibition of cancer cells is caused by repression of MAPK, AKT, STAT signaling pathways after drug therapy. Besides, silencing PDGFR could result in vessel atrophy and reduce energy source from glycolysis. Abnormal lipid accumulation acts as an alternative energy source and provides fuels for survival of tumor cells. Tumor cells proliferate independent of classical signaling pathways and therefore exhibit drug resistance [37].

In this study, we identified that LINC00160 was associated with sunitinib resistance. RCC cells highly expressed LINC00160 to recruit transcriptional factor-TFAP2A, which bound to SAA1 promoter regions and activated its expression. On one hand, SAA1 anchored to ABCB1 protein, which facilitated sunitinib cellular efflux and diminished drug accumulation. On the other hand, SAA1 stimulated JAK-STAT signaling pathways, which countered cellular survival inhibition from drug. All these regulatory networks were well organized and collaborated, thus promoting sunitinib resistance in renal cell carcinoma.

## MATERIALS AND METHODS

### Cell culture

The 786-O and ACHN human RCC cell lines were purchased from the American Type Culture Collection (Manassas, VA, USA). Cells were maintained at 37°C in a 5% CO_2_ incubator and cultured in high glucose Dulbecco's modified Eagle's medium (Thermo Fisher Scientific, Inc., Waltham, MA, USA), containing 10% fetal bovine serum (Thermo Fisher Scientific, Inc.) and 1% penicillin-streptomycin. Sunitinib resistant cell lines ACHN-R and 786-O-R were established by exposing ACHN and 786-O to an initial dose of sunitinib (2 μM) and gradually increasing concentrations up to 5 μM [27].

### Separation of nuclear and cytoplasmic RNA assay

Separation of nuclear and cytoplasmic RNA was performed using PARIS kit (Thermo Fisher Scientific, Inc.) according to the manufacturer’s instruction. GAPDH and U6 were used as cytoplasmic and nuclear control separately.

### RT-qPCR

Total RNA was extracted using TRIzol reagent (Thermo Fisher Scientific, Inc.). RNAs were reverse-transcribed to cDNA using a PrimeScript RT reagent Kit (Takara Biotechnology Co., Ltd., Dalian, China). RT-qPCR was performed using the ABI StepOnePlus system (Thermo Fisher Scientific, Inc.). Relative expression was calculated using the -2^ΔΔCt^ method, and GAPDH was used as the internal control. U6 primers were synthesized by Ribobio (Guangzhou, China) and other primers were listed in the Supplementary Table 1.

### Cell infection and transfection

Expression lentivirus for LINC00160 short hairpin RNA (LINC00160 shRNA) (Vigene Biosciences, USA) and negative control (Vigene Biosciences, USA) were transfected in ACHN-R and 786-O-R cell lines at a multiplicity of infection (MOI) of 15 assisted with ADV-HR (Vigene Biosciences, USA) according to the manufacturer’s instructions. For plasmids transfections, cells were cultured in six well plants and transfected with 4 μg expression plasmids for LINC00160 shRNA (Vigene Biosciences, USA), Ctrl shRNA (Vigene Biosciences, USA), LINC00160 (Genechem, China), Vector (Genechem, China), SAA1 shRNA (Vigene Biosciences, USA), Ctrl shRNA (Vigene Biosciences, USA), SAA1 (Vigene Biosciences, USA) and Vector (Vigene Biosciences, USA) using Lipofectamine 2000 (Thermo Fisher Scientific, Inc.) according to the manufacturer’s instructions. Small interfering RNA (siRNA) (TranSheepBio, China) for TFAP2A was transfected in ACHN-R and 786-O-R cell lines using Lipofectamine 2000 (Thermo Fisher Scientific, Inc.) according to the manufacturer’s instructions.

### Cell counting kit-8 (CCK-8) assay

Cells were seeded into 96-well plates at a density of 1000 cells per well. CCK-8 reagents (Djingo, Japan) were added into wells at 24, 48, 72, 96 hours. The optical density was measured at a wavelength of 450 nm.

### Cell viability assay

After transfection, cells were seeded into 96-well plates at a density of 1000 cells per well and treated with sunitinib at indicated concentration for 48 hours. Cell viability was measured with CCK-8 reagents at a wavelength of 450 nm.

### Transwell assay

Cell invasion assays were performed using 8 μm pore-size chambers coated with 60 μl matrigel gel (Corning, Inc., NY, USA). 2 × 10^4^ cells were resuspended in serum-free medium and seeded into the upper chambers; the bottom chambers were filled with medium containing serum. After incubation for 24 hours, the invasive cells were stained and imaged under 100X magnification. Five random fields were analyzed for each chamber. Cell migration assays were performed using chambers without matrigel gel. 1.5 × 10^4^ cells were resuspended in serum-free medium and other procedures were the same as above.

### Colony formation

Thousand cells per well were seeded and cultured for 2 weeks. The plates were fixed with methanol and stained with crystal violet. Cells were imaged and counted for the amounts.

### Wound healing assay

When confluence reached 90% in 6-well plates, a 10 μl pipette tip was used to scratch on the monolayer. Then, cells were washed and starved to migrate for indicated time. Images were taken at 0, 24, 36 hours under 40X magnification.

### Flow cytometry analysis of cell cycle

Cells were trypsinized in chilled PBS, fixed in 70% cold ethanol overnight. After washed with chilled PBS, cells were stained with propidiumiodide (Beyotime, China). Samples were analyzed with a FACS-Calibur Flow Cytometer (BD Biosciences, San Jose, CA, USA) using CellQuest Pro software (BD Biosciences, San Jose, CA, USA).

### Western blotting (WB)

RIPA lysis buffer (Servicebio, China) with phosphorylation protease inhibitor (Servicebio, China) were used to extract the protein of cells. The proteins were separated by gel electrophoresis and transferred to polyvinylidene fluoride membranes (Roche, Basel, Switzerland). Membranes were blocked with 5% nonfat milk or bovine serum albumin (BSA) for 2 hours at room temperature and incubated with primary antibodies in 4 °C overnight. The membranes were washed and then incubated with secondary antibodies for 2 hours. The antibodies used for Western blotting were listed in the Supplementary Table 2.

### Immunoprecipitation (IP)

RIPA lysis buffer (Servicebio, China) with phosphorylation protease inhibitor (Servicebio, China) were used to extract the protein of cells. Protein A-sepharose beads were washed by RIPA and centrifuged at 1,000 × g in a micro centrifuge to remove supernatant. Cell lysis buffer was mixed with beads and primary antibodies against the specific antigen were added. The mix was incubated with rotation overnight at 4 °C to form the immunocomplex and the step proceeded for Western blotting assay. Total proteins (input controls) and normal rabbit IgG controls were simultaneously assayed. The antibodies used for IP were listed in the Supplementary Table 2.

### RNA immunoprecipitation (RIP)

RIP assays were conducted using the Magna RIP™ RNA-Binding Protein Immunoprecipitation Kit (Millipore, USA). The procedures were followed according to the manufacturer’s instructions. Analysis of immunoprecipitated RNA was conducted by quantitative RT-PCR. Total RNA (input controls) and normal rabbit IgG controls were simultaneously assayed. The primers used for detecting LINC00160 are listed in the Supplementary Table 1. The antibodies used for RIP were listed in the Supplementary Table 2.

### Luciferase assay

Cells were co-transfected with pGL3-basic-SAA1 dual-luciferase reporter (Vigene Biosciences, USA), phRL-Renilla-luciferase (Vigene Biosciences, USA), TFAP2A siRNA (TranSheepBio, China) or negative control (TranSheepBio, China) using Lipofectamine 2000 (Thermo Fisher Scientific, Inc.). Cells were plated in 24-well plates and collected to detect luciferase activity using Dual-Luciferase® Reporter Assay System (Promega, Madison, WI, USA) 48 hours after transfection. Transfection efficiency was normalized by Renilla luciferase activity.

### Chromatin immunoprecipitation assay and promoter analysis (CHIP)

CHIP assays were conducted using SimpleChIP® Enzymatic Chromatin IP Kit (Agarose Beads) #9002 (CST, USA). The procedures were followed according to the manufacturer’s instructions. Primers designed for specific promoter regions of SAA1 were listed in the Supplementary Table 3.

### *In vivo* xenograft and treatment experiments

6-week old male athymic BALB/c nude mice were obtained from Vital River Laboratory Animal Technology (Beijing, China). 7 x 10^6^ 786-O-R cells were injected subcutaneously into the flanks of nude mice. Tumor size was measured weekly and xenograft volume was calculated by the formula: tumor volume (mm^3^) = longer diameter × shorter diameter^2^/2. When the volume reached approximately 200 mm^3^, mice were orally administrated with vehicle or sunitinib (40 mg/kg/day) once a day for 4 weeks. When mice were killed, tumors were weighed and paraffin-embedded for immunohistochemistry assays. Animal experiments were approved by the Institutional Animal Use and Care Committee of Tongji Medical College, Huazhong University of Science and Technology (HUST).

### Immunohistochemistry (IHC)

Tumor samples were fixed in 4% paraformaldehyde, permeabilized with 0.5% TritonX-100for and blocked with 5% goat serum. Samples were incubated with primary antibodies at 4 °C overnight and detected with HRP-conjugated secondary antibody for 1 hour at room temperature. IHC was performed using antibodies against p-ERK1/2 (Abcam, ab201015); p-AKT1 (Abcam, ab81283); p-STAT3 (Abcam, ab76315); c-PARP1 (Abcam, ab32064).

### Correlation analysis and GO analysis

Correlation analysis of the expression of LINC00160 in kidney cancer samples was conducted on Excel 2016 using the CORREL function. Mapping genes of LINC00160 with a correlation coefficient of >0.5 were selected. Data from the functional enrichment analysis of the biological process and molecular function Gene Ontology (GO) terms were downloaded from STRING. P-value<0.05 was considered statistically significant. Enrichment plots were created by imageGP (http://www.ehbio.com/ImageGP/index.php/Home/Index/GOenrichmentplot.html).

### Gene set enrichment analysis (GSEA)

GSEA (http://www.broadinstitute.org/gsea) as performed on KEGG gene sets collected from the TCGA-KIRC database. Gene expression profiles were ranked from high to low and divided into two groups, according to candidate gene expression level. For enriched gene sets, FDR<25% and nominal P<0.05 were considered to be statistically significant. Venn diagrams were created using Draw Venn Diagram (http://bioinformatics.psb.ugent.be/webtools/Venn/).

### Bioinformatics prediction

Transcriptional factors were from GeneCards (https://www.genecards.org/) and PROMO (http://alggen.lsi.upc.es/cgi-bin/promo_v3/promo/promoinit.cgi?dirDB=TF_8.3).

### Statistics

Statistical analysis was carried out using Graphpad Prism 7.0 software, and each experiment was performed at least three times. The Student's *t*-test was used to analyze differences in the gene expression between two groups. Data is presented as the mean ± SEM, and P<0.05 was considered to indicate a statistically significant difference.

### Ethics statement

This study was approved by the Ethics Committees of Huazhong University of Science and Technology, and all aspects of the study comply with the criteria established by the Declaration of Helsinki.

## Supplementary Material

Supplementary Figures

Supplementary Tables
