# Supplementary material for: LINC00160 mediates sunitinib resistance in renal cell carcinoma via SAA1 that is implicated in STAT3 activation and compound transportation
Source: Aging (Albany NY). 2020 Sep 13;12(17):17459–79. doi: 10.18632/aging.103755 (PMC7521490; doi:10.18632/aging.103755)
Supplement: Supplementary Figures [file aging-12-103755-s002..pdf]

SUPPLEMENTARY FIGURES

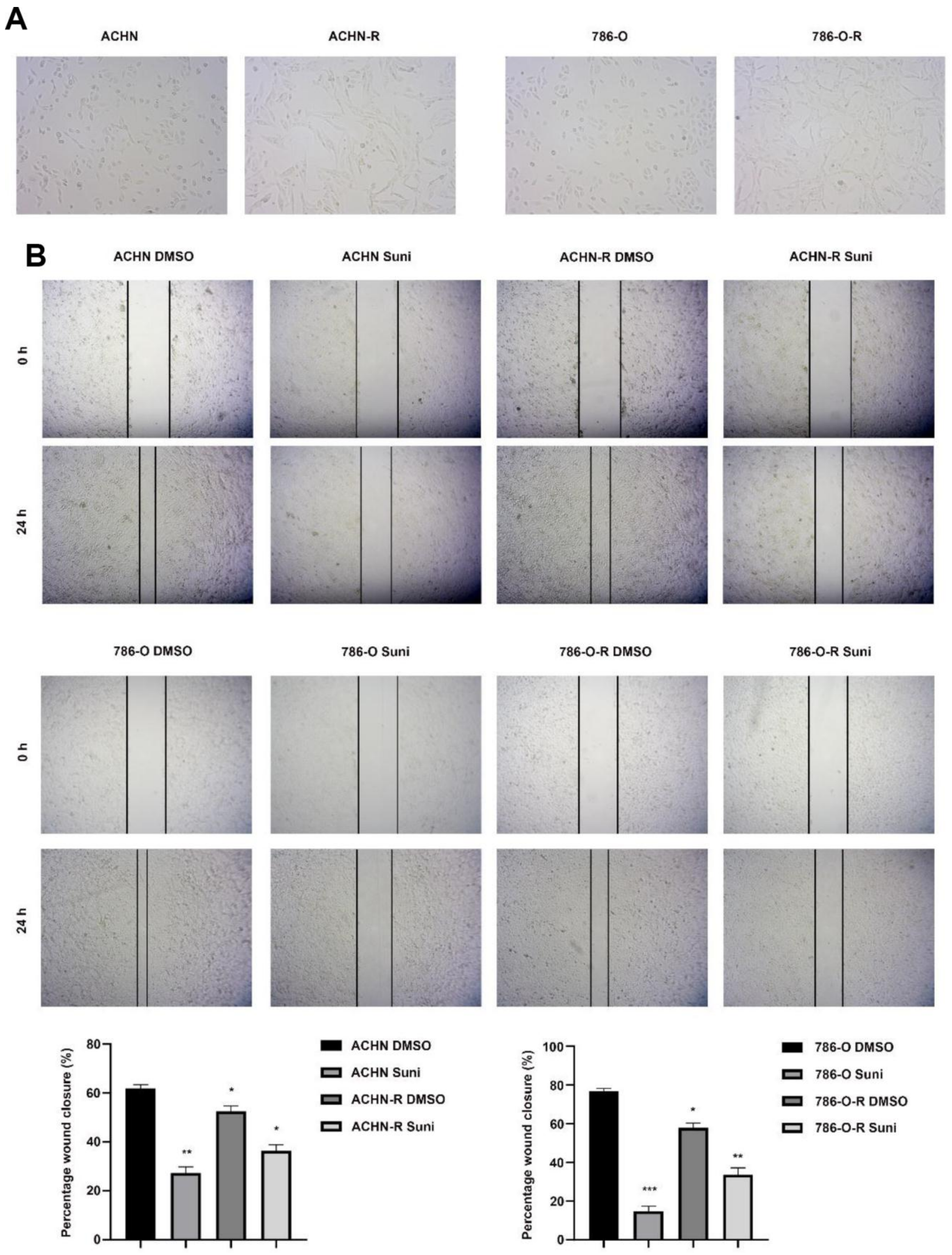

Supplementary Figure 1. Comparison between parental and resistant cells. (**A**) Cell morphology between parental cells ACHN, 786-O and resistant cells ACHN-R, 786-O-R. (**B**) Wound healing assays of resistant and parental cells with/without sunitinib treatment. Each experiment was performed at least three times and data was represented as mean ± SEM. *P<0.05, **P<0.01 and ***P<0.001.

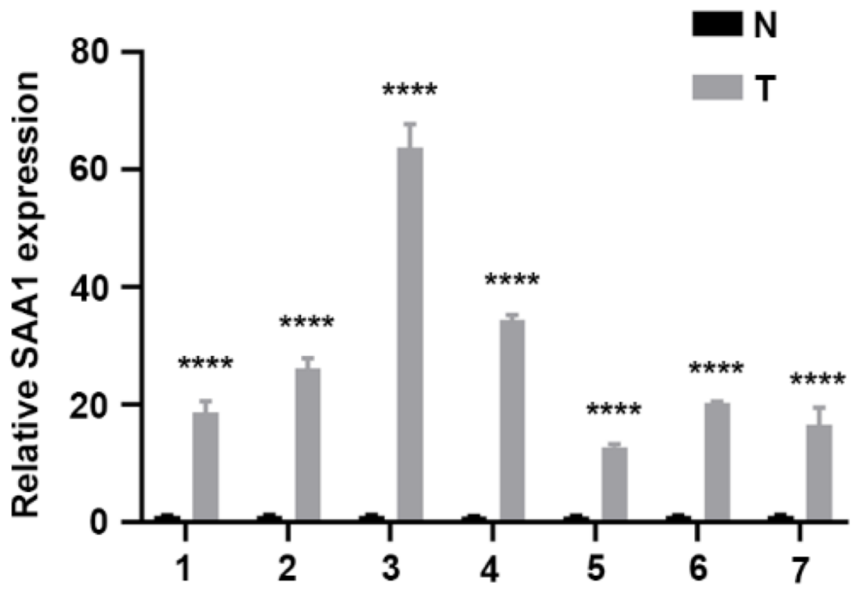

Supplementary Figure 2. SAA1 is overexpressed in clinical samples. Each experiment was performed at least three times and data was represented as mean ± SEM. *P<0.05, **P<0.01, ***P<0.001 and ****P<0.0001.

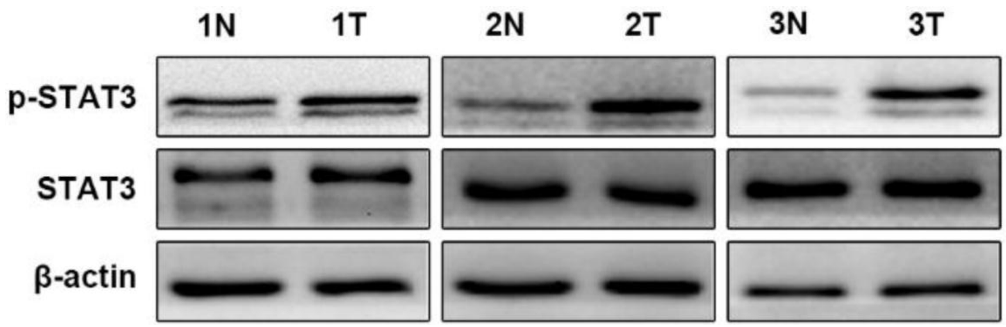

Supplementary Figure 3. JAK-STAT3 signaling pathway is activated in clinical samples. Each experiment was performed at least three times.

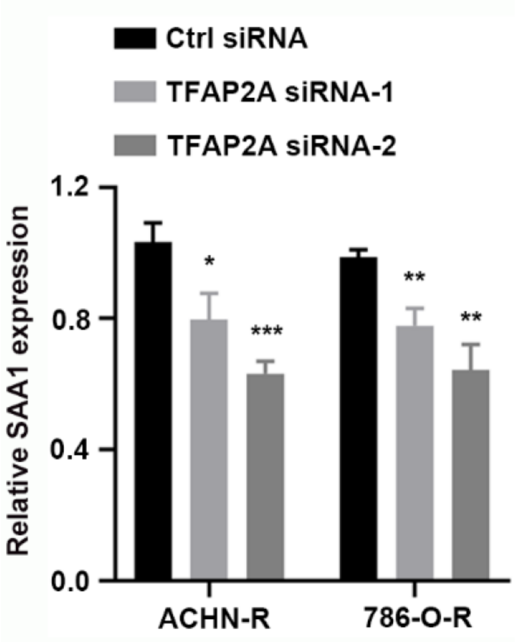

Supplementary Figure 4. Lower expression of SAA1 after TFAP2A knockdown. Each experiment was performed at least three times and data was represented as mean ± SEM. *P<0.05, **P<0.01, ***P<0.001.
