# Supplementary material for: LINC00160 mediates sunitinib resistance in renal cell carcinoma via SAA1 that is implicated in STAT3 activation and compound transportation
Source: Aging (Albany NY). 2020 Sep 13;12(17):17459–79. doi: 10.18632/aging.103755 (PMC7521490; doi:10.18632/aging.103755)
Supplement: Supplementary Tables [file aging-12-103755-s001..pdf]

SUPPLEMENTARY TABLES

Supplementary Table 1. Primers used for RT-qPCR for indicated genes.

Gene	Forward (5'-3')	Reverse (5'-3')
GAPDH	AAAAGCATCACCCGGAGGAGAA	AAGGAAATGAATGGGCAGCCG
LINC00160	ACAGCCAACCACCCATTCTCTT	AGGGAAGGCAGCAGACAAAACC
SAA1	ACAGCACAGATCAGCACCATGA	AGAAGCTTCGGCTGCTGACA
ABCB1	GGGAGCTTAACACCCGACTTA	GCCAAAATCACAAGGGTTAGCTT
TFAP2A	CTCCGCCATCCCTATTAACAAG	GACCCGGAACTGAACAGAAGA
C16orf74	CCGTCCTGAACGACAAGCAC	TCAGGCTTCTGGGTCGATTTC
IL20RB	CATCCTTACCCGACCTGGGA	ACGAATGTCTGGGCCTTCAC
IL1R2	GACACTACGCACCACAGTCA	CCAGTTCTGTGTTTGCACCG
F2	TGCAGAGATCGGCATGTCAC	TCGGTGAAGTTCTTGTCCCAG
C1R	CAGCCTCAGTACCAGTTCCG	CACTCCCATTGTGGTGGTGT
TGFBI	GTGCGGCTAAAGTCTCTCCA	CATGGACCACGCCATTTGTG
LBP	TCAGCCCTGGGAATCTGTCT	AAGGTGGCGGACACATTAGT
RARRES1	CACTACTACTTGGCACAGCTCA	GGAGGCTTCTTCTGGTGTCTG

Supplementary Table 2. Antibodies used for this research.

Antibody	Cat. Number	Company
p-ERK1/2	ab201015	Abcam
ERK1/2	ab184699	Abcam
p-AKT1	ab81283	Abcam
AKT1	ab179463	Abcam
p-STAT3	ab76315	Abcam
STAT3	ab68153	Abcam
c-PARP1	ab32064	Abcam
β-actin	AC004	ABclonal
ABCB1	A11747	ABclonal
	22336-1-AP	Proteintech
SAA1	A1655	ABclonal
TFAP2A	13019-3-AP	Proteintech
IgG	AC005	ABclonal
	B900610	Proteintech

Supplementary Table 3. Primers used for ChIP-PCR for indicated fragments.

base pair sites	Forward (5'-3')	Reverse (5'-3')
-2000/-1600	TATGAACTTCTGGCTGGGCA	TGCATCTCAACCTCCCAAGT
-1442/-1309	GAGCCATGATTGCACCACTG	ACTCCTCACCTGATCCTCCT
-819/-810	TGCTGCTATATCCACCAGCCTCT	CTCAGCACAGAAAAGCCCTGCTC
-770/-400	GAGTGCAGTGGCGTGAT	GCCAAGGCAGGAGGATC
-360/-164	TGGCTTCAAAGCTGCCCTCAC	CAGCTCTGCTCCTCAGCATTGA
